# Metabolic Dysfunction in Alzheimer’s Disease: Brain Glucose Hypometabolism as an Early Precursor to Amyloid and Tau Pathology

**DOI:** 10.3390/jcm15051884

**Published:** 2026-03-01

**Authors:** Rafail C. Christodoulou, Daniel Eller, Platon S. Papageorgiou, Efthalia Angelopoulou, Evros Vassiliou, Sokratis G. Papageorgiou

**Affiliations:** 1 Division of Neuroimaging and Neurointervention, Department of Radiology, Stanford University, Stanford, CA 94304, USA; deller@stanford.edu; 2 Department of Medicine, Medical School, National and Kapodistrian University of Athens, 11527 Athens, Greece; platopap@med.uoa.gr; 3 1st Department of Neurology, Medical School, National and Kapodistrian University of Athens, Eginition Hospital, 15772 Athens, Greece; angelthal@med.uoa.gr (E.A.); sokpapa@med.uoa.gr (S.G.P.); 4 Department of Biological Sciences, Kean University, Union, NJ 07083, USA; evassili@kean.edu

**Keywords:** Alzheimer’s disease, type 3 diabetes, proteinopathies, metabolic failure, glucose metabolism, Apoε4

## Abstract

**Objective:** Alzheimer’s disease (AD) is traditionally characterized by amyloid-β and tau pathology; however, accumulating evidence indicates that metabolic and inflammatory dysfunctions are early, central contributors to disease development. This narrative review explores how metabolic disturbances influence AD pathophysiology. **Methods:** A comprehensive literature search was performed on PubMed, Embase, and Scopus. Selected studies were original studies or reviews published in English within the past five years involving human subjects. Case reports, case series, editorials, and non-human studies were excluded. A total of 64 articles were reviewed and summarized. **Results:** Cerebral glucose hypometabolism, mitochondrial impairment, insulin resistance, oxidative stress, and neuroinflammation were observed throughout the AD spectrum. These metabolic changes often appeared before significant amyloid accumulation and were more closely linked to tau pathology and cognitive decline. Early microglial activation was linked to transient glucose hypermetabolism, progressing to glucose hypometabolism and neurodegeneration as the disease advanced. **Conclusions:** AD is associated with a gradual breakdown of metabolic and inflammatory homeostasis, which occurs before and promotes the development of traditional neuropathological features. Addressing early metabolic vulnerabilities may be essential for effective disease intervention and prevention.

## 1. Introduction

Alzheimer’s disease (AD) is characterized by neuropathological features, including the buildup of extracellular amyloid-β (Aβ) plaques and the presence of intracellular neurofibrillary tangles composed of hyperphosphorylated tau (p-tau) [1,2]. Both Aβ and p-tau are implicated in the drive of synaptic loss, neurodegeneration, and cognitive decline. However, the amyloid cascade hypothesis seems to be the most dominant in neurodegeneration research, positing that the amyloid byproduct is the key initiator of downstream tau accumulation [3]. Although this cascade has revealed crucial insights, it has not yet translated into fully effective therapies. Indeed, although several large clinical trials have demonstrated promising results for antiamyloid therapies that achieve strong amyloid plaque clearance but do not yield corresponding cognitive improvements, they have prompted a critical re-examination of the amyloid-centered model [4]. The amyloid mechanistic cascade has been criticized by multiple AD researchers, who argue that the amyloid-tau pedigree is insufficient to account for the complex pathophysiology of sporadic AD [4]. Therefore, a possible shift in other complementary mechanisms is now observed. In particular, the brain metabolic dysfunction hypothesis appears crucial in driving, and even preceding, protein-related aggregations in AD [5].

Several studies have already suggested that AD is not solely a disease of misfolded proteins but also involves impaired energy metabolism [6]. Consistent postmortem and in vivo studies demonstrate that the AD brain is in an energy-deficient state, characterized by decreased glucose utilization and reduced cerebral metabolic activity [7]. Notably, Fluorodeoxyglucose PET imaging reveals hypometabolism in vulnerable regions even in individuals with mild cognitive impairment or preclinical AD, often before extensive Aβ plaque deposition is evident [8]. These findings suggest that glucose underutilization may be an early finding in AD rather than just a result of neurodegeneration. Clinical evidence links metabolic disorders to AD risk, with insulin resistance and type 2 diabetes patients more prone to developing AD, called Type 3 diabetes due to shared pathogenic features. Disruption of insulin and glucose pathways impairs neuronal energy metabolism and promotes Aβ and tau accumulation [7,9]. Therefore, metabolic dysfunction, including glucose hypometabolism, insulin signaling defects, mitochondrial dysfunction, and oxidative stress, is increasingly recognized as a central factor in AD development rather than a peripheral aspect [7,9].

Metabolic dysfunction appears to occur early in the disease process. Initially, cerebral glucose hypometabolism deprives neurons of their main energy source. Since normal brain function requires substantial energy, a persistent deficit in glucose use can make neurons and synapses more susceptible to damage and hasten their degeneration [8,9,10]. This phenomenon involves brain insulin resistance, in which AD brains exhibit decreased insulin signaling, leading to reduced glucose uptake and metabolism in neurons [9]. Eventually, resistance impairs neuronal glucose use and worsens Aβ and tau pathologies, as reduced insulin/IGF signaling and energy sensing diminish Aβ clearance and increase tau phosphorylation [11]. Genetic factors, such as APOE ε4 and familial AD mutations, are associated with impaired brain glucose transport and utilization. These findings imply that brain hypometabolism and disturbances in the insulin pathway may trigger neuropathological changes [11]. During energy cell utilization, mitochondrial dysfunction is a hallmark of AD, characterized by impaired oxidative phosphorylation and reduced ATP levels in AD neurons, resulting in energy deficits and elevated reactive oxygen species (ROS) [12].

Oxidative damage and reduced cellular antioxidant defenses are closely associated with the accumulation of Aβ and tau, highlighting oxidative stress as a key factor in AD [12]. Interestingly, Aβ oligomers further worsen the cascade by impairing mitochondrial function and elevating ROS levels, forming a feedback loop linking metabolic failure to neuronal damage [13]. Research across molecules, animal models, and humans has reinforced the point that energy shortages accelerate the buildup of amyloid and tau. Specifically, reduced glucose availability alters amyloid precursor protein (APP) processing, promoting Aβ production, while insulin resistance activates kinases such as glycogen synthase kinase-3β (GSK-3β), thereby increasing tau phosphorylation [14]. These statements have also been supported by neuroimaging, which shows that regions of hypometabolism align with areas rich in Aβ and tau, and that decreased glucose metabolism is associated with cognitive decline [15].

Overall, metabolic dysfunction drives AD and is interconnected with amyloid and tau pathology. Understanding these metabolic problems could lead to biomarkers and treatments such as mitochondrial protectants, insulin therapies, or antioxidants that target early disease mechanisms and improve outcomes. This review synthesizes insights into the metabolic origins of AD and their connections to amyloid and tau pathology. By combining molecular, imaging, and clinical data, it provides clinicians and researchers with a detailed understanding of AD, supporting the hypothesis that the disease may originate from cellular metabolic dysfunction years before symptoms emerge.

## 2. Materials and Methods

The methodological principles of this structured narrative review were guided by the Scale for the Assessment of Narrative Review Articles (SANRA) recommendations [16]. An academic medical librarian (D.E.) conducted a literature search using PubMed, Embase, and Scopus to identify studies on metabolic dysfunction in AD during August–November 2025. The search combined controlled keywords related to cerebral metabolism, mitochondrial dysfunction, insulin resistance, inflammation, Alzheimer’s disease, tau, and amyloid. Detailed search strategies for each database are included in the Appendix A.

This review primarily focused on English-language publications involving human subjects published within the past five years, reflecting the rapid growth of research on metabolic dysfunction in Alzheimer’s disease. Earlier seminal studies were included when necessary to provide a mechanistic context and foundational insights. Only original research articles and review papers were considered. At the same time, case reports, case series, editorials, letters, conference abstracts, and non-human studies were excluded to emphasize clinical relevance and high-quality evidence. Though this was a structured narrative review, special focus was placed on longitudinal cohort studies, multimodal imaging, particularly those combining Fluorodeoxyglucose-Positron Emission Tomography (FDG-PET) with amyloid or tau biomarkers, and studies with biomarker-confirmed Alzheimer’s disease diagnoses. When available, larger multicenter cohorts and studies using standardized diagnostic criteria were prioritized over small exploratory analyses. Cross-sectional studies were included if they offered mechanistic insights or supported stage-specific metabolic findings, but longitudinal data were deemed more informative for understanding the temporal relationship between metabolic dysfunction and proteinopathies. Due to the variability in study designs and outcomes, findings were integrated thematically rather than quantitatively.

Records were screened by titles and abstracts for relevance, with full-text review as needed. Irrelevant studies and duplicates were removed before final selection. Owing to the diversity in methodologies, biomarkers, and imaging techniques, no quantitative analysis was conducted. Instead, findings were summarized thematically to explore common mechanisms linking metabolic dysfunction, inflammation, and AD. In all, 64 articles were included in the final narrative review.

## 3. Results

The literature review identified 64 suitable articles, including original human studies and review articles focusing on metabolic and inflammatory changes in AD. Key findings from these studies include the following:

- Consistent cerebral glucose hypometabolism in temporoparietal and posterior cingulate regions across the AD spectrum, which correlates more strongly with cognitive decline and tau pathology than with amyloid-β levels.
- Mitochondrial dysfunction and oxidative stress are frequently connected to neuronal damage, synaptic failure, and tau buildup, often emerging in preclinical or early symptomatic phases. Insulin resistance and impaired glucose regulation, both central and peripheral, are linked to faster cognitive decline and higher levels of tau-related biomarkers.
- Neuroinflammation shows a stage-dependent pattern, with early microglial activation linked to metabolic hyperactivity, and later stages marked by hypometabolism and neurodegeneration. Peripheral immune markers, such as altered neutrophil-to-lymphocyte ratios, are associated with brain metabolic changes, tau pathology, and structural atrophy.

Overall, these findings suggest that metabolic and inflammatory dysregulation are early and fundamental aspects of AD pathology, rather than mere secondary effects of amyloid accumulation.

## 4. Discussion

### 4.1. Metabolic Dysfunction as an Upstream and Relevant Driver of Neurodegeneration

Growing evidence from genetic, sporadic, systemic, and phenotypic variants of AD indicates that cerebral hypometabolism and broader metabolic dysfunction are central to neurodegeneration [17]. For instance, recent evidence from Down syndrome—related AD provides a strong example of a metabolism-first approach, as widespread cerebral hypometabolism is present even in asymptomatic individuals and advances in a temporoparietal pattern that closely resembles that seen in sporadic AD [18]. Hypometabolism in this genetic variant of AD was more strongly associated with the cerebrospinal fluid neurofilament light chain (NfL), a marker of axonal damage, than with amyloid or tau biomarkers, suggesting that metabolic decline may be a better indicator of neuronal vulnerability and injury than traditional protein markers; it also implies that metabolic dysfunction could indicate ongoing neurodegeneration before the accumulation of amyloid or tau becomes dominant [18].

Longitudinal data on AD patients emphasize metabolism as an early risk marker for the disease. In a large cohort, a normal 18FDG-PET scan showed a high negative predictive value for short-term dementia and neurodegeneration biomarkers, effectively ruling out imminent progression in individuals without dementia [19]. This supports the idea that maintained cerebral glucose metabolism reflects neuronal resilience, while hypometabolism may serve as an early indicator of neurodegeneration.

In addition to glucose metabolism, systemic metabolic stressors appear to have both independent and combined effects on brain health. Elevated homocysteine levels and diabetes mellitus jointly worsen brain atrophy, without increasing amyloid or tau deposits [20]. The disconnection between metabolic toxicity and typical AD proteinopathies indicates that metabolic harm can cause structural and functional brain damage through mechanisms partly separate from amyloid and tau, questioning a solely protein-focused view of neurodegeneration [21]. Spatial imaging studies further clarify the relationship between metabolism and protein aggregation. Multimodal PET analyses demonstrate that hypometabolism and tau deposition show strong regional overlap in the temporoparietal cortices, whereas amyloid deposition is more diffuse and less tightly linked to clinical severity [21,22]. Both hypometabolism and tau increased monotonically with worsening cognitive impairment and neuropsychiatric symptoms, whereas amyloid burden plateaued earlier, highlighting metabolism and tau as closer correlates of clinical dysfunction [23]. Advanced voxel-wise metabolic—tau ratio imaging further refines this relationship, revealing that in preclinical and prodromal stages, glucose hypometabolism and tau deposition may initially occur in distinct regions before converging spatially in later stages of disease [24], suggesting that metabolic decline can precede or evolve independently of tau pathology during early disease phases, before becoming tightly coupled as neurodegeneration progresses. Importantly, metabolic vulnerability differs among AD phenotypes. In posterior cortical atrophy, early-onset cases mostly display parietal hypometabolism, whereas late-onset cases often involve more temporal and limbic regions, resembling typical AD [25].

Indeed, the metabolic aspect is notably complex, especially in cases of mixed proteinopathies. In patients with both AD and Lewy body disease, significant posterior hypometabolism was found, which exceeded what could be explained by regional atrophy and tau levels [26]. The metabolic mismatch was also associated with reduced cerebrospinal fluid levels of dopamine metabolites and synaptic markers, suggesting that impaired neurotransmission and neuronal health are key factors in metabolic decline [26]. These deficits go beyond what structural imaging or tau-PET can detect, highlighting hypometabolism as a sensitive marker of overall cellular stress driven by multiple pathological processes.

**Figure 1:**
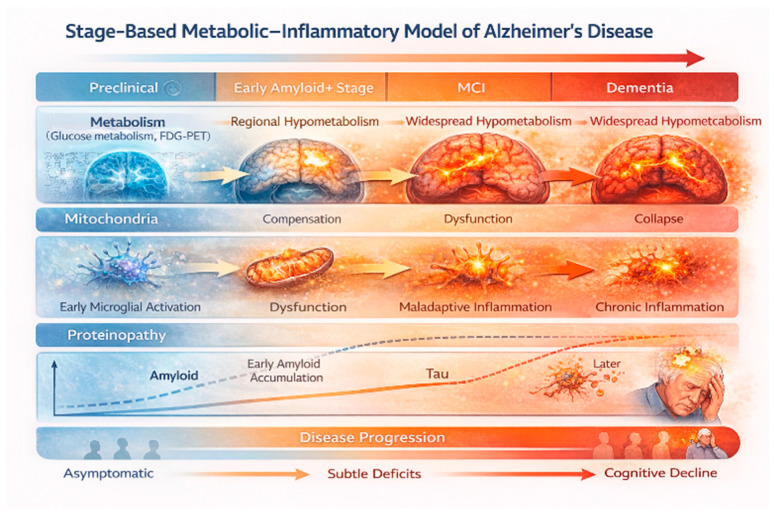
Stage-based Metabolic–Inflammatory Model of Alzheimer’s Disease. Alzheimer’s disease is depicted as a progressive disorder driven by early metabolic and inflammatory vulnerability. Initial stages are characterized by subtle metabolic alterations and microglial activation with preserved or compensatory mitochondrial function. As the disease advances, regional glucose hypometabolism, mitochondrial dysfunction, maladaptive neuroinflammation, and tau pathology increasingly align with clinical impairment. Amyloid accumulation appears early and plateaus, showing weaker associations with metabolic decline and cognitive severity. Created in BioRender. Christodoulou, R. (2026) https://BioRender.com/3ljimlk.

AD is considered a highly complex disease with multiple contributing factors to its pathophysiology and progression. This complexity is evident when molecular and systemic metabolic data also indicate early metabolic vulnerability. Insulin resistance has been linked to faster cognitive decline in non-demented adults, although its association with long-term changes in amyloid or tau biomarkers remains weak [11,14]. At a more detailed level, soluble oligomeric Aβ, but not fibrillar plaque burden, correlates with prolonged glucose dysregulation in individuals without clear tau pathology [27]. This suggests that early metabolic toxicity caused by soluble amyloid species is a key component of the Type 3 diabetes hypothesis. Importantly, this effect appears to depend on tau stage, supporting the idea that metabolic dysfunction, amyloid toxicity, and tau pathology interact dynamically across different disease stages (Figure 1) [27].

Overall, metabolic dysfunction is a major, varied contributor to neurodegeneration in AD. Rather than a straightforward amyloid cascade, AD features an extended period of cellular metabolic stress shaped by genetics, systemic factors, homeostasis, phenotype, and co-pathologies. This stress weakens neuronal resilience, promoting proteinopathies and cognitive decline. The major metabolic and inflammatory contributors to neurodegeneration across the AD continuum are summarized in Table 1.

### 4.2. Metabolic–Mitochondrial Dysfunction as a Mechanistic Link Between Glucose Hypometabolism, Neuroinflammation, and Amyloid–Tau Pathology

Experimental, imaging, and biomarker evidence indicates that metabolic dysfunction in AD arises from mitochondrial dysfunction, endoplasmic reticulum (ER) stress, insulin resistance, and oxidative injury. These factors create an environment conducive to amyloid and tau neurodegeneration [6,28]. They are interconnected yet time-dependent, with metabolic issues often preceding or occurring independently of tau and amyloid aggregation.

Evidence of metabolic dysfunction supports therapeutic modulation studies. For example, in the PEGASUS trial, sodium phenylbutyrate and taurursodiol, which target mitochondrial and proteostatic stress, reduced CSF tau, markers of synaptic degeneration, gliosis, and inflammatory mediators, despite limited short-term clinical effects [29]. The trial’s findings suggest that mitochondrial, inflammatory, and neurodegenerative pathways are involved, supporting the view that metabolic and organellar stress are primary drivers of proteinopathy rather than just secondary effects. Additionally, changes in oxidative stress markers highlight a redox imbalance within this metabolic cascade [29]. Because we need direct metabolic biomarkers, these help clarify the relationship further, as reduced CSF lactate levels, an indirect measure of cerebral metabolic activity, were found in AD and frontotemporal dementia but not in dementia with Lewy bodies. They showed an inverse correlation with tau but not amyloid biomarkers specifically within the AD spectrum [10,30]. This suggests that amyloid pathology alone may not be sufficient to cause neuronal metabolic failure. In contrast, tauopathy and neuronal injury are more closely associated with impaired energy metabolism, supporting a model where metabolic decline and tau pathology are mechanistically linked.

**Figure 2:**
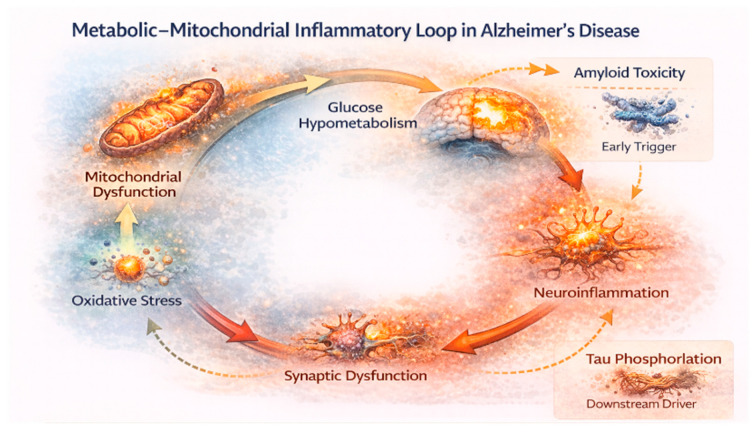
Metabolic–Mitochondrial–Inflammatory Feedback Loop in Alzheimer’s Disease. A self-reinforcing cycle links mitochondrial dysfunction, glucose hypometabolism, oxidative stress, neuroinflammation, and synaptic dysfunction, driving progressive neurodegeneration. Tau pathology amplifies this loop and closely aligns with metabolic decline, while amyloid acts as an early trigger but is insufficient to sustain neurodegeneration on its own. Created in BioRender. Christodoulou, R. (2026) https://BioRender.com/6t2ql5z.

There is an urgent need to develop methods to gather more information about the mitochondrial microenvironment, given its crucial role in neurodegeneration [31]. Advances in mitochondrial imaging have increased the level of detail available. PET using mitochondrial complex I tracers revealed a strong spatial link between mitochondrial dysfunction and glucose hypometabolism, as well as domain-specific cognitive impairment, but consistently failed to identify regional connections with amyloid burden [32,33]. Notably, mitochondrial complex I dysfunction was negatively correlated with tau deposition in early Braak regions, supporting the idea that mitochondrial impairment is more closely associated with tau-mediated neuronal injury than with fibrillar amyloid accumulation (Figure 2) [33]. The importance of mitochondrial dysfunction in AD may extend beyond the brain. Recent studies show that better mitochondrial function in skeletal muscle correlates with a significantly lower risk of developing mild cognitive impairment or dementia, decreased amyloid PET uptake, and reduced plasma neuroinflammatory markers [34]. This systemic link suggests that mitochondrial health reflects a general metabolic condition that affects both brain and body risk factors for neurodegeneration, connecting brain-specific changes to the overall homeostatic status of body energy metabolism.

Regarding mitochondria and overall cell dysfunction, it might be helpful to analyze things in more detail, starting with possible synaptic dysfunction in neurons caused by various factors. At the synapse, initial compensatory mechanisms seem to accelerate in early stages but eventually break down. Higher levels of CSF neuronal pentraxin-2 (NPTX2), a marker of synaptic plasticity, were positively correlated with glucose metabolism in the precuneus during early mild cognitive impairment, indicating a temporary period of metabolic and synaptic resilience at the beginning of the disease [35]. As the disease advances, declining NPTX2 levels align with worsening hypometabolism and cognitive decline, pointing to the failure of the brain’s adaptive metabolic responses [35,36].

Building on cell dysfunction and neuronal networks, synaptic dysfunction is also involved. Insulin resistance is a central factor in this mechanistic network. Studies demonstrating increased brain glucose uptake via GLP-1 signaling show restoration of cerebral metabolism, reduced amyloid levels, decreased neuroinflammation, and reduced tau hyperphosphorylation [37]. A hypothesis was tested in a Phase 2b clinical trial (ELAD), in which patients with mild-to-moderate AD received liraglutide. The trial used FDG-PET to assess the primary outcome, the change in cerebellar glucose metabolic rate. The results showed that there was no statistically significant difference in cerebral glucose metabolism between groups over the time of 52 weeks in the 204 participants, even though improved performance on the executive cognitive test, ADAS-Exec, was observed, but no significant benefit was observed in daily living activities, as measured by the ADAS-ADL [38]. Specifically, early preliminary data from EVOKE and EVOKE+ trials in 2025 that have been presented in major AD scientific conferences explained in detail that first, there is no significant benefit of the oral form of semaglutide versus placebo in the improvement of cognition and function domains as measured by Clinical Dementia Rating—Sum of Boxes(CDR-SB) or in reducing the disease progression. They also show that despite the lack of clinical improvement, there was an improvement in the biomarker profile of the patients. These clinical trials represent a major milestone in AD research, as they enrolled 3808 patients with mild cognitive impairment or mild dementia over 156 weeks and improved our understanding of the multifactorial nature of AD [39].

Although neither the ELAD nor the EVOKE/EVOKE+ trials showed strong clinical benefits on primary cognitive measures, their results remain insightful within a metabolic-inflammatory view of AD. The lack of notable cognitive improvement, despite indications in metabolic or biomarker areas, may be due to the complexity and stage-specific nature of disease progression. In a condition marked by prolonged metabolic vulnerability and network dysfunction, focusing solely on insulin signaling or glucose use might not be enough once irreversible synaptic loss has happened. These trials emphasize an important point discussed in this manuscript: metabolic treatments are likely most effective when given early, during the preclinical or prodromal stages, before extensive hypometabolism and neurodegeneration set in. Additionally, the disconnect between biomarker changes and clinical outcomes underscores the multifaceted nature of AD, suggesting that combined approaches such as metabolic stabilization, anti-inflammatory strategies, and protein-targeted therapies may be necessary for meaningful disease modification. Rather than dismissing the metabolic hypothesis, these findings refine it, underscoring the importance of patient stratification, timing of interventions, and the use of metabolic engagement markers in future trials. Conversely, further human research suggests that insulin resistance, combined with low CSF Aβ42 levels, worsens tau pathology, although insulin resistance alone does not directly cause tau accumulation [28]. These findings imply that metabolic stress heightens neuronal vulnerability to tau-related damage when early amyloid abnormalities are present.

Finally, oxidative stress and neuroinflammation amplify metabolic dysfunction [12]. Human postmortem studies further indicate that translocator protein (TSPO)-positive microglial activation is closely linked to tau, but not to amyloid, in vulnerable cortical areas, along with increased levels of inflammatory cytokines, such as interleukin-15 [40]. These findings support broader models connecting mitochondrial dysfunction, oxidative stress, and neuroinflammation as mutually reinforcing factors in AD progression [41]. The mechanistic framework shows that mitochondrial dysfunction, impaired glucose metabolism, insulin resistance, oxidative stress, and neuroinflammation interact to promote tau pathology and neuron damage, with amyloid as an early but insufficient trigger. This pathway explains the long preclinical phase of AD and suggests an early intervention target.

### 4.3. The Cellular Microenvironment and the Decades-Long Preclinical Phase of Alzheimer’s Disease

Increasing evidence from human research indicates that AD develops within a gradually changing cellular environment long before visible amyloid plaques, tau tangles, or clinical signs appear [42]. Instead of a rapid pathological event, AD seems to result from persistent, low-level metabolic and inflammatory disruptions that alter neuronal—glial relationships over many years [43]. The preclinical stage of AD includes microglial activation, immune system imbalance, and metabolic shifts in glial cells, which together create conditions favorable for future neurodegeneration [44,45].

Specifically, microglial activation is among the earliest detectable responses to emerging AD pathology. Longitudinal studies of CSF and imaging reveal that triggering receptor expressed on myeloid cells 2 (TREM2)-related microglial responses closely align with the initial stages of amyloid fibrilization, even before significant plaque buildup becomes apparent. In individuals with early amyloid positivity (CSF Aβ+/PET−), an increase in amyloid burden correlates with higher CSF sTREM2 and p-tau181 levels, as well as FDG-PET hypermetabolism, which likely reflects inflammatory activation rather than sustained neuronal function, suggesting that early microglial activity is energy-consuming and potentially maladaptive [46]. As the disease progresses, this relationship changes; in later amyloid stages, higher soluble triggering receptor expressed on myeloid cells 2 (sTREM2) levels are linked to glucose hypometabolism and tau accumulation, signaling a shift from inflammatory hyperactivity to metabolic decline [46,47], suggesting a stage-specific microglial course in which early immune responses initially promote tau pathology and eventually drive neurodegeneration. Compensatory hypermetabolism in the context of microglial activation refers to transient increases in regional glucose uptake, as seen on FDG-PET images, that occur early during microglial activation and synaptic stress. During this phase, structural integrity and network connectivity remain mostly intact. The heightened metabolic activity likely reflects inflammatory energy demands rather than efficient neuronal function. Conversely, pathological hypometabolism involves persistent decreases in glucose utilization linked to synaptic loss, mitochondrial dysfunction, tau buildup, and network disconnection, and it is closely associated with cognitive decline impairment [46,47].

Importantly, neuroinflammatory processes extend beyond the central nervous system. Large-scale human studies show that peripheral immune balance is closely connected to cognition, brain metabolism, and key AD biomarkers. For instance, increased neutrophil counts and a higher neutrophil-to-lymphocyte ratio are associated with reduced overall cognition, FDG-PET hypometabolism, ventricular enlargement, and elevated CSF tau. Conversely, higher lymphocyte levels correlate with maintained metabolism, less brain atrophy, and more favorable amyloid and tau profiles [48]. Mediation analyses reveal that these immune effects on cognition are partly driven by amyloid and tau pathology, emphasizing a two-way relationship between systemic immunity and neurodegeneration [49,50]. Findings like these could support the idea that chronic peripheral immune activation influences the brain environment long before clinical dementia appears.

Research in immunology focusing on metabolism indicates that reprogramming glial cell metabolism could be an early step in AD development [50]. When reactive astrocytes encounter inflammatory signals, they undergo significant metabolic changes, including increased glycolysis [51]. Astrocytes in similar environments can exhibit rapid, rhythmic fluctuations in metabolic intermediates, detectable at the single-cell level, that may precede irreversible neuronal damage [51]. Although these findings are from controlled, human-relevant cellular models, they suggest that early metabolic disturbances in glial cells may initiate later synaptic and neuronal failures rather than merely being a consequence of other disease processes.

The pattern of neuroinflammation in the brain underscores its role in the progression of AD [52]. In vivo TSPO-PET imaging shows that microglial activation primarily occurs along highly connected brain networks, mirroring the connectivity-based spread observed with tau pathology [40]. Higher microglial activation in anterior medial temporal areas correlates with worse cognitive performance and dementia severity, indicating that inflammation targets vulnerable brain network areas [40], which suggests that metabolic and inflammatory stress may spread through functional pathways, gradually affecting large brain network systems over time.

The AD model begins with long-term disturbances in the cellular environment, such as early immune activation, glial metabolic dysfunction, and systemic inflammation [53]. These interact to cause chronic metabolic stress, making neural networks more vulnerable to amyloid and tau buildup, suggesting that protein aggregation is a consequence, not the cause, of cellular vulnerability. We should view AD as a disorder of the cellular environment, emphasizing the preclinical phase as a key target for intervention and underscoring the importance of strengthening metabolic and inflammatory resilience before symptoms develop. A stage-based conceptual framework integrating metabolic, inflammatory, and proteinopathic processes across the AD continuum is presented in Table 2.

### 4.4. Metabolic-First and Amyloid-First Perspectives: Toward Integration Rather than Opposition

Two main hypothesis models have shaped AD research. The amyloid-first model, based on genetic and neuropathological data, posits that Aβ accumulation is the initial trigger, leading to tau pathology, synaptic dysfunction, and neurodegeneration. This hypothesis has guided many therapeutic efforts and is supported by genetic studies of familial AD and amyloid imaging [54]. Conversely, the metabolic-first model highlights early bioenergetic dysfunction, insulin resistance, and impaired glucose utilization as initial contributors. Studies show that metabolic syndrome and insulin signaling dysfunction are linked to accelerated Aβ accumulation and cognitive decline and that metabolic dysfunction can precede or exacerbate characteristic protein deposits [55]. These models are not mutually exclusive. Metabolic issues can promote amyloid production and mitochondrial dysfunction, whereas amyloid and tau pathologies can impair neuronal metabolism and increase oxidative stress, creating a cycle of energy deficiency and protein stress [56]. Overall, rather than viewing metabolic and amyloid processes as opposing, recent evidence suggests they interact synergistically. This integrated perspective views metabolic vulnerability and amyloid pathology as interconnected factors jointly driving the complex progression of AD.

### 4.5. Clinical Implications

This review’s evidence suggests reinterpreting AD as a disorder rooted in longstanding metabolic and inflammatory vulnerabilities, rather than being caused solely by amyloid-β or tau buildup. In all forms of AD, whether genetic, sporadic, or phenotypically diverse, disturbances in glucose metabolism, mitochondrial processes, immune signaling, and interactions between glial cells and neurons appear early and change over decades. Under this view, amyloid and tau are less the initial triggers and more the downstream effects of a persistently altered cellular environment.

This approach helps resolve several longstanding inconsistencies in AD research. It explains why amyloid levels alone poorly correlate with cognitive decline, why tau and brain hypometabolism align more closely with clinical symptoms, and why therapies targeting amyloid have shown limited success. Importantly, it indicates that effective disease-modifying strategies should focus on conditions that enable proteinopathies to become toxic, rather than on clearing proteins once neurodegeneration has begun.

Clinically, this emphasis on metabolic and inflammatory processes opens new avenues for early detection and intervention. Biomarkers reflecting brain metabolism, mitochondrial health, microglial activity, and systemic immune status could be especially valuable during the preclinical and early symptomatic stages, when neuronal networks still retain some neuroplasticity. These markers could complement existing amyloid and tau frameworks by identifying at-risk individuals before irreversible brain damage occurs. Importantly, this framework supports a precision-medicine approach in which patients are stratified by metabolic—inflammatory phenotype (e.g., insulin resistance, T2D status, APOE genotype, vascular burden, inflammatory signatures) rather than by amyloid positivity alone. Such stratification may improve trial enrichment and help match interventions to the dominant upstream driver in a given individual.

From a practical standpoint, an implementation pathway could involve early risk detection using clinical or metabolic profiling and sensitive cognitive or behavioral screening, confirmation and staging with multimodal biomarkers (FDG-PET or other metabolic measures, plus amyloid and tau as appropriate), and longitudinal monitoring of metabolic and inflammatory markers to guide escalation or combination therapy. It is worth mentioning that interpreting FDG-PET findings in Alzheimer’s disease requires accounting for potential confounders. Factors such as age-related metabolic decline, cerebrovascular disease, systemic metabolic conditions (e.g., hyperglycemia and insulin resistance) during scanning, medication effects, and cortical atrophy can affect regional glucose uptake. Additionally, neuroinflammatory activation may temporarily raise FDG signals independent of neuronal function. These considerations highlight the importance of combining FDG-PET results with structural imaging, biomarker data, and clinical information to accurately identify stage-specific metabolic changes.

Therapeutically, this model advocates for shifting toward approaches that strengthen metabolic resilience and restore cellular homeostasis in the brain. Treatments targeting insulin pathways, mitochondrial function, oxidative stress, and maladaptive neuroinflammation may be most effective when applied early and, if possible, combined with amyloid- or tau-focused therapies (Table 3). Additionally, lifestyle and systemic factors such as metabolic factors, vascular risk management, and chronic stress should be considered intrinsic parts of AD pathology rather than merely disease modifiers. A key implication is the need for metabolic target-engagement endpoints in clinical trials, such as normalization or stabilization of regional FDG-PET patterns, mitochondrial function readouts where available, and shifts in inflammatory biomarkers, alongside traditional amyloid and tau measures. These endpoints could provide earlier signals of biological efficacy than cognitive outcomes, which often lag behind underlying pathophysiology. However, real-world translation will require scalable, low-cost biomarker strategies (e.g., blood-based markers and digital phenotyping) to complement higher-cost imaging, enabling earlier detection beyond specialized centers.

### 4.6. Limitations and Future Directions

This review has several limitations. First, much of the current evidence remains correlational, making it challenging to establish definitive causal relationships between metabolic dysfunction, inflammation, and protein aggregation in AD. Second, the scope of the included literature was restricted to English-language publications from the past five years and studies conducted exclusively in human populations, while case reports were excluded. Limiting inclusion to the most recent five years may have led to the underrepresentation of foundational earlier work that established key concepts and methodological advances in brain energy metabolism and AD. However, this time restriction was applied deliberately because the literature in this field has expanded rapidly in recent years, driven by advances in biomarker science, multimodal neuroimaging (e.g., FDG-PET combined with amyloid/tau PET), and emerging metabolic and inflammatory measures. Hence, focusing on the latest evidence helps capture the current state of the art and the most clinically actionable insights. These selection criteria, although chosen to enhance relevance and translational value, may have introduced selection and publication bias. Future research should prioritize long-term, longitudinal, and multimodal human studies to better delineate temporal relationships among metabolic, inflammatory, and proteinopathic processes and identify critical therapeutic windows for early intervention.

APOE ε4, the strongest genetic risk factor for sporadic AD, also aligns closely with the “metabolic-first” framing of AD [57]. FDG-PET studies show that cognitively normal APOE ε4 homozygotes may exhibit reduced cerebral glucose metabolism in AD-vulnerable regions in late midlife, providing “preclinical” metabolic evidence of AD risk [57]. Mechanistically, APOE4 may amplify metabolic vulnerability by perturbing neuronal-glial energy coupling and insulin signaling. For example, experimental work suggests that APOE4 can impair neuronal insulin receptor signaling pathways, which are tightly linked to glucose utilization and downstream tau-related stress responses [58]. Future directions should prioritize longitudinal, genotype-stratified (and sex-stratified) multimodal studies that integrate FDG-PET with tau and amyloid biomarkers, as well as emerging mitochondrial and inflammation imaging, and test whether early metabolic interventions (e.g., insulin-pathway modulation) might normalize hypometabolism and alter downstream tau-associated neurodegeneration in APOE4 carriers.

Circadian rhythm disruption is increasingly recognized as part of the broader metabolic phenotype of AD [57]. Clinically, AD is often accompanied by sleep-wake fragmentation and “sundowning”, consistent with a weakened central clock and impaired coordination of energy demand across neural networks [59]. Mechanistically, circadian clocks regulate cellular energetics and glucose handling. In AD models, amyloid-β has been shown to disrupt core clock machinery by degrading the brain and muscle ARNT-like protein 1 (BMAL1) and CREB-binding protein (CBP), providing a plausible route by which proteotoxic stress can drive circadian misalignment and, secondarily, metabolic inefficiency [60]. Complementary evidence links circadian disturbance to energetic failure within the suprachiasmatic nucleus (SCN), the master pacemaker [61]. Future directions should also standardize time-of-day and sleep-state documentation in fluorodeoxyglucose positron emission tomography (FDG-PET) and other metabolic readouts to reduce circadian confounding and test chronobiology-informed interventions, such as bright light, melatonin, timed feeding, or exercise, specifically for their ability to stabilize circadian organization and preserve regional glucose metabolism in at-risk populations.

Future directions should also consider mild behavioral impairment (MBI) as a practical, symptom-based framework for linking early neuropsychiatric changes to the metabolic cascade of AD. Given that MBI captures later-life emergent, persistent neuropsychiatric symptoms that may precede overt cognitive decline, future longitudinal studies should test whether MBI status and domains (e.g., apathy, affective dysregulation, impulse dyscontrol) predict regional brain glucose hypometabolism on FDG-PET and whether these metabolic signatures mediate subsequent tau accumulation and cognitive trajectories [62]. Integrating MBI phenotyping with multimodal biomarkers (FDG-PET, amyloid and tau PET, plasma and CSF markers, and inflammatory measures such as sTREM2, where feasible) could help clarify temporal ordering and identify a clinically accessible “behavioral-at-risk” subgroup for early metabolic intervention trials [63]. Finally, interventional studies should evaluate whether targeting metabolic dysfunction improves MBI symptoms and alters downstream neurodegeneration, thereby positioning MBI as both an early enrichment marker and a meaningful, patient-centered outcome in prevention-oriented AD research.

Therapeutically, the evidence synthesized in this review supports a shift toward metabolism- and inflammation-targeted strategies that intervene upstream of, or in parallel with, amyloid and tau accumulation. Potential approaches include enhancing brain insulin signaling and glucose utilization (e.g., intranasal insulin and other insulin-pathway modulators), improving mitochondrial bioenergetics and reducing oxidative stress (mitochondria-directed agents and redox-modulating strategies), and attenuating maladaptive neuroinflammatory activation (microglia-focused interventions guided by inflammatory biomarkers [64]. In parallel, multidomain lifestyle interventions, such as structured physical activity, dietary optimization, and sleep and circadian stabilization, remain promising as low-risk methods to improve systemic and cerebral metabolic resilience. Critically, future clinical trials should incorporate metabolic and inflammatory biomarkers, such as FDG-PET, mitochondrial and inflammation PET when available, and blood or CSF markers, as target-engagement readouts and stratify participants by metabolic phenotype (e.g., insulin resistance, APOE genotype, baseline hypometabolism) to identify subgroups most likely to benefit and define the optimal therapeutic window for early intervention.

Monoclonal antibodies targeting amyloid should also be interpreted within this metabolic-inflammatory framework as interventions that primarily reduce protein burden but may not fully address the upstream “vulnerability terrain” that enables proteinopathies to become clinically toxic. This helps explain why substantial plaque removal has not always translated into proportional cognitive benefit. Downstream clearance can occur while synaptic energetics, mitochondrial function, and glia—neuron metabolic coupling remain impaired. Conversely, these agents may be most effective when deployed early, before widespread network hypometabolism and irreversible neurodegeneration, and when combined or sequenced with strategies that restore metabolic resilience and modulate maladaptive neuroinflammation. In this view, monoclonal antibodies serve as one component of a broader disease-modifying strategy, and future trials should integrate metabolic and inflammatory target-engagement markers to identify responders, optimize timing, and clarify whether improving the cellular milieu amplifies the clinical impact of protein-targeted clearance.

## 5. Conclusions

In summary, AD should be seen not just as a disorder caused by Aβ and tau buildup but as a result of prolonged metabolic and inflammatory imbalance that precedes classical neuropathology. Brain glucose metabolism, mitochondrial function, insulin signaling, and immune regulation are often altered decades before symptoms appear. These changes alter the cellular environment, causing neurons and glia to endure chronic stress and low-grade inflammation, promoting protein aggregation and neurodegeneration. In this context, amyloid and tau act more as amplifiers of an already vulnerable system than as primary causes, explaining the weak link between amyloid levels and cognition and the stronger link between tau and metabolic decline with clinical symptoms. This view emphasizes the roles of glial cells and systemic factors, such as immune balance and metabolic health, in disease progression. Clinically, seeing AD as a disorder of metabolic and inflammatory resilience highlights the need for early detection beyond protein biomarkers, including measures of metabolic and immune health. It also supports early, combined treatments aimed at restoring cellular balance instead of just removing proteins after degeneration. Recognizing AD as a gradual failure of metabolic and inflammatory homeostasis frames the preclinical phase as a vital window for intervention and offers a comprehensive model that links molecular, cellular, systemic, and network dysfunction to better prevent and treat AD.

## Figures and Tables

**Table 1:** Metabolic and Inflammatory Drivers of Neurodegeneration Across the Alzheimer’s Disease Continuum.

Domain	Key Findings	Pathophysiological Implications	Reference
Cerebral glucose metabolism	Early and progressive cerebral hypometabolism precedes dementia and predicts neurodegeneration. Normal FDG-PET has high negative predictive value.	Hypometabolism reflects early neuronal vulnerability and loss of metabolic resilience.	[17,18,19]
Genetic and phenotypic AD variants	Down syndrome-related AD and posterior cortical atrophy show early region-specific hypometabolism.	Metabolic failure is a shared upstream feature across AD subtypes.	[18,25]
Tau pathology	Regional overlap between glucose hypometabolism and tau deposition. Both may correlate with clinical severity.	Tau-mediated neurodegeneration is tightly coupled to metabolic decline.	[21,22,23,24]
Amyloid pathology	Amyloid deposition is diffuse, plateaus early, and weakly correlates with metabolism and cognition.	Amyloid acts as an early trigger but is insufficient to explain neurodegeneration.	[21,22,23]
Systemic metabolic stress	Diabetes, hyperhomocysteinemia, and insulin resistance worsen atrophy and cognition independent of amyloid or tau.	Systemic metabolic toxicity contributes directly to brain injury.	[11,14,20]
Mixed proteinopathies	AD with Lewy body pathology shows disproportionate hypometabolism beyond tau or atrophy.	Metabolic dysfunction integrates multiple pathological insults.	[26]
Soluble amyloid toxicity	Oligomeric Aβ correlates with glucose dysregulation in early disease stages.	Early metabolic toxicity occurs before overt tau accumulation.	[27]

**Table 2:** Conceptual Model of Alzheimer’s Disease as a Disorder of Metabolic and Inflammatory Homeostasis.

Disease Stage	Dominant Cellular Processes	Metabolic—Inflammatory Features	Clinical or Biomarker Manifestations	Reference
Preclinical	Subtle glial activation, metabolic reprogramming, immune imbalance	Microglial activation, astrocytic glycolytic shifts, peripheral immune dysregulation	FDG-PET hypermetabolism, elevated sTREM2, and immune system biomarkers	[42,43,44,45,46,48,49,50,51]
Early amyloid-positive stage	Inflammatory energy demand exceeds metabolic compensation	Transition from hypermetabolism to regional hypometabolism	Rising p-tau, altered FDG-PET patterns	[46,47]
Prodromal AD (MCI)	Mitochondrial dysfunction, insulin resistance, synaptic stress	Decline in glucose metabolism and synaptic plasticity markers	Reduced NPTX2, regional hypometabolism	[35,36]
Dementia stage	Metabolic collapse, tau-driven neurodegeneration	Widespread hypometabolism, oxidative stress, chronic inflammation	FDG-PET hypometabolism, cognitive decline	[21,22,23,24,40,41]
Network-level propagation	Pathology spreads along vulnerable functional networks	Microglial activation and metabolic stress follow connectivity pathways	TSPO-PET network-based inflammation	[40,52]
Systemic contribution	The brain reflects the whole-body metabolic state	Mitochondrial health in peripheral tissues modulates neurodegeneration risk	Lower dementia risk with preserved systemic mitochondrial function	[35,53]

Abbreviations: AD, Alzheimer’s disease; FDG-PET, fluorodeoxyglucose positron emission tomography; MCI, mild cognitive impairment; NPTX2, neuronal pentraxin-2; p-tau, phosphorylated tau; sTREM2, soluble triggering receptor expressed on myeloid cells 2; TSPO-PET, translocator protein positron emission tomography.

**Table 3:** Stage-Specific Metabolic Interventions Across the Alzheimer’s Disease Continuum.

Disease Stage	Dominant Metabolic and Inflammatory Features	Representative Biomarkers	Proposed Metabolic Interventions	Rationale
Preclinical (Aβ+/PET−)	Microglial activation, FDG hypermetabolism, early insulin dysregulation	sTREM2 ↑, FDG hypermetabolism, peripheral immune markers	Anti-inflammatory modulation, lifestyle metabolic optimization, circadian stabilization	Reduce maladaptive immune activation and metabolic stress before tau coupling
Early Amyloid-Positive	Transition from hyper- to hypometabolism of glucose	Rising p-tau, regional FDG decline	Insulin pathway modulators (intranasal insulin, GLP-1 agents)	Restore neuronal glucose utilization and prevent tau acceleration
Prodromal (MCI)	Mitochondrial dysfunction and synaptic stress	Reduced NPTX2, FDG hypometabolism, CSF lactate changes	Mitochondrial stabilizers, redox-targeted agents	Improve bioenergetic resilience and slow tau-driven degeneration
Dementia Stage	Widespread hypometabolism, chronic inflammation	Marked FDG decline, tau-PET burden	Combination strategies (metabolic and protein-targeted therapies)	Address both downstream proteinopathy and upstream metabolic collapse

## Data Availability

No new data were generated.

## Appendix A

Search Strategy Tables

### Appendix A.1. PubMed (MEDLINE)

**Set #**	**Concept**	**Syntax**	**Results**
1	Alzheimer spectrum (population)	(“Alzheimer Disease” [MeSH Terms] OR Alzheimer * [Title/Abstract] OR “AD dementia” [Title/Abstract] OR “mild cognitive impairment” [Title/Abstract] OR MCI [Title/Abstract] OR “prodromal Alzheimer *” [Title/Abstract])	264,559
2	Metabolic & bioenergetic dysfunction	(“Brain” [MeSH Terms] OR “Energy Metabolism” [MeSH Terms] OR “Glucose Metabolism Disorders” [MeSH Terms] OR brain metabolism [Title/Abstract] OR cerebral metabolism [Title/Abstract] OR glucose hypometabolism [Title/Abstract] OR insulin resistance [Title/Abstract] OR mitochondrial dysfunction [Title/Abstract] OR oxidative stress [Title/Abstract])	2,722,287
3	Imaging (PET)	(“Positron-Emission Tomography” [MeSH Terms] OR PET [Title/Abstract] OR FDG-PET [Title/Abstract] OR 18F-FDG [Title/Abstract])	176,691
4	Amyloid pathology	(“Amyloid beta-Peptides” [MeSH Terms] OR amyloid beta [Title/Abstract] OR amyloid accumulation [Title/Abstract] OR amyloid depositionfi [Title/Abstract])	140,007
5	Tau pathology	(“tau Proteins” [MeSH Terms] OR tau [Title/Abstract] OR tau pathology [Title/Abstract] OR neurofibrillary tangle * [Title/Abstract])	79,228
6	Limits/Filters	English; Humans; Publication date ≥ 2021; Original research and reviews only	
7	Full combined query	1 AND 2 AND 3 AND 4 AND 5 AND 6 = ((“Alzheimer Disease” [MeSH Terms] OR “alzheimer *” [Title/Abstract] OR “AD dementia” [Title/Abstract] OR “mild cognitive impairment” [Title/Abstract] OR “MCI” [Title/Abstract] OR “prodromal alzheimer *” [Title/Abstract]) AND (“Brain” [MeSH Terms] OR “Energy Metabolism” [MeSH Terms] OR “Glucose Metabolism Disorders” [MeSH Terms] OR “brain metabolism” [Title/Abstract] OR “cerebral metabolism” [Title/Abstract] OR “cerebral glucose metabolism” [Title/Abstract] OR “brain glucose” [Title/Abstract] OR “hypometabolism” [Title/Abstract] OR “glucose hypometabolism” [Title/Abstract] OR “glucose hypometaboli *” [Title/Abstract] OR “energy failure” [Title/Abstract] OR “brain energy failure” [Title/Abstract] OR “energy metabolism failure” [Title/Abstract] OR “metabolic dysfunction” [Title/Abstract] OR “metabolic dysfunct *” [Title/Abstract] OR “Insulin Resistance” [MeSH Terms] OR “Insulin Resistance” [Title/Abstract] OR “brain insulin resistance” [Title/Abstract] OR “central insulin resistance” [Title/Abstract] OR “insulin receptor *” [Title/Abstract] OR “brain insulin receptor *” [Title/Abstract] OR “type 3 diabetes” [Title/Abstract] OR “Mitochondria” [MeSH Terms] OR “mitochondria *” [Title/Abstract] OR “mitochondrial dysfunction” [Title/Abstract] OR “mitochondrial bioenergetics” [Title/Abstract] OR “ATP depletion” [Title/Abstract] OR “Oxidative Stress” [MeSH Terms] OR “Oxidative Stress” [Title/Abstract] OR “glutathione” [Title/Abstract] OR “reactive oxygen species” [Title/Abstract] OR “ROS” [Title/Abstract] OR “redox imbalance” [Title/Abstract]) AND (“Positron-Emission Tomography” [MeSH Terms] OR “PET” [Title/Abstract] OR “FDG-PET” [Title/Abstract] OR “18F-FDG” [Title/Abstract] OR “fluorodeoxyglucose” [Title/Abstract]) AND (“Amyloid beta-Peptides” [MeSH Terms] OR “amyloid beta” [Title/Abstract] OR “Abeta” [Title/Abstract] OR “Abeta” [Title/Abstract] OR “amyloid accumulation” [Title/Abstract] OR “amyloid deposition” [Title/Abstract]) AND (“tau Proteins” [MeSH Terms] OR “tau” [Title/Abstract] OR “tau pathology” [Title/Abstract] OR “neurofibrillary tangle *” [Title/Abstract] OR “paired helical filament *” [Title/Abstract]) AND (“2021/01/12 00:00”:”3000/01/01 05:00” [Date-Publication] AND “humans” [MeSH Terms] AND “english” [Language] AND (“all” [Filter] NOT “preprint” [Publication Type])) AND (“2021/01/13 00:00”:”3000/01/01 05:00” [Date-Publication] AND (“all” [Filter] NOT “preprint” [Publication Type]) AND “humans” [MeSH Terms] AND “english” [Language])) AND (adaptiveclinicaltrial [Filter] OR classicalarticle [Filter] OR clinicalstudy [Filter] OR clinicaltrial [Filter] OR introductoryjournalarticle [Filter] OR meta-analysis [Filter] OR multicenterstudy [Filter] OR networkmetaanalysis [Filter] OR pragmaticclinicaltrial [Filter] OR randomizedcontrolledtrial [Filter] OR review [Filter] OR scientificintegrityreview [Filter] OR scopingreview [Filter] OR systematicreview [Filter] OR validationstudy [Filter])	92

### Appendix A.2. Embase

**Set #**	**Concept**	**Syntax**	**Results**
1	Alzheimer spectrum (population)	(‘alzheimer disease’/exp OR alzheimer *: ti,ab OR ‘ad dementia’: ti,ab OR ‘mild cognitive impairment’/exp OR mci: ti,ab)	418,807
2	Metabolic & bioenergetic dysfunction	(‘brain metabolism’/exp OR ‘glucose metabolism’/exp OR hypometabolism: ti,ab OR insulin resistance: ti,ab OR mitochondrial dysfunction: ti,ab OR oxidative stress: ti,ab)	437,658
3	Imaging (PET)	(‘positron emission tomography’/exp OR pet: ti,ab OR ‘fdg pet’: ti,ab)	401,181
4	Amyloid pathology	(‘amyloid beta’/exp OR ‘amyloid accumulation’: ti,ab OR ‘amyloid deposition’: ti,ab)	98,943
5	Tau pathology	(‘tau protein’/exp OR tau: ti,ab OR ‘neurofibrillary tangle *’: ti,ab)	99,831
6	Limits/Filters	English; Humans; Publication year ≥ 2021; Article or Review	
7	Full combined query	1 AND 2 AND 3 AND 4 AND 5 AND 6 = (‘alzheimer disease’/exp OR ‘alzheimer disease’ OR alzheimer *: ti,ab OR ‘ad dementia’: ti,ab OR ‘mild cognitive impairment’/exp OR ‘mild cognitive impairment’ OR ‘mild cognitive impairment’: ti,ab OR mci: ti,ab OR ‘prodromal alzheimer *’: ti,ab) AND (‘brain metabolism’/exp OR ‘brain metabolism’ OR ‘glucose metabolism’/exp OR ‘glucose metabolism’ OR ‘energy metabolism’/exp OR ‘energy metabolism’ OR ‘brain metabolism’: ti,ab OR ‘cerebral metabolism’: ti,ab OR ‘cerebral glucose metabolism’: ti,ab OR ‘brain glucose’: ti,ab OR hypometabolism: ti,ab OR ‘glucose hypometabolism’: ti,ab OR ‘glucose hypometaboli *’: ti,ab OR ‘energy failure’: ti,ab OR ‘brain energy failure’: ti,ab OR ‘energy metabolism failure’: ti,ab OR ‘metabolic dysfunction’: ti,ab OR ‘metabolic dysfunct *’: ti,ab OR ‘insulin resistance’/exp OR ‘insulin resistance’ OR ‘insulin resistance’: ti,ab OR ‘brain insulin resistance’: ti,ab OR ‘central insulin resistance’: ti,ab OR ‘insulin receptor’/exp OR ‘insulin receptor’ OR ‘insulin receptor *’: ti,ab OR ‘brain insulin receptor *’: ti,ab OR ‘type 3 diabetes’: ti,ab OR ‘mitochondrion’/exp OR ‘mitochondrion’ OR mitochondria *: ti,ab OR ‘mitochondrial dysfunction’: ti,ab OR ‘mitochondrial bioenergetics’: ti,ab OR ‘atp depletion’: ti,ab OR ‘oxidative stress’/exp OR ‘oxidative stress’ OR ‘oxidative stress’: ti,ab OR glutathione: ti,ab OR ‘reactive oxygen species’: ti,ab OR ros: ti,ab OR ‘redox imbalance’: ti,ab) AND (‘positron emission tomography’/exp OR ‘positron emission tomography’ OR pet: ti,ab OR ‘fdg pet’: ti,ab OR ‘18f fdg’: ti,ab OR fluorodeoxyglucose: ti,ab) AND (‘amyloid beta’ OR ‘amyloid beta’: ti,ab OR abeta: ti,ab OR aβ: ti,ab OR ‘amyloid accumulation’: ti,ab OR ‘amyloid deposition’: ti,ab) AND (‘tau protein’/exp OR ‘tau protein’ OR tau: ti,ab OR ‘tau pathology’: ti,ab OR ‘neurofibrillary tangle *’: ti,ab OR ‘paired helical filament *’: ti,ab) AND (2020py OR 2021:py OR 2022:py OR 2023:py OR 2024:py OR 2025:py OR 2026:py) AND (‘alzheimer disease’/dm/exp OR ‘alzheimer disease’) AND (‘clinical article’/exp OR ‘clinical article’ OR ‘clinical trial’/exp OR ‘clinical trial’ OR ‘clinical trial topic’/exp OR ‘clinical trial topic’ OR ‘cohort analysis’/exp OR ‘cohort analysis’ OR ‘comparative study’/exp OR ‘comparative study’ OR ‘computer model’/exp OR ‘computer model’ OR ‘controlled study’/exp OR ‘controlled study’ OR ‘cross sectional study’/exp OR ‘cross sectional study’ OR ‘diagnostic test accuracy study’/exp OR ‘diagnostic test accuracy study’ OR ‘double blind procedure’/exp OR ‘double blind procedure’ OR ‘human’/exp OR ‘human’ OR ‘longitudinal study’/exp OR ‘longitudinal study’ OR ‘major clinical study’/exp OR ‘major clinical study’ OR ‘meta analysis’/exp OR ‘meta analysis’ OR ‘multicenter study’/exp OR ‘multicenter study’ OR ‘observational study’/exp OR ‘observational study’ OR ‘phase 1 clinical trial topic’/exp OR ‘phase 1 clinical trial topic’ OR ‘phase 2 clinical trial’/exp OR ‘phase 2 clinical trial’ OR ‘phase 2 clinical trial topic’/exp OR ‘phase 2 clinical trial topic’ OR ‘phase 3 clinical trial’/exp OR ‘phase 3 clinical trial’ OR ‘phase 3 clinical trial topic’/exp OR ‘phase 3 clinical trial topic’ OR ‘randomized controlled trial’/exp OR ‘randomized controlled trial’ OR ‘randomized controlled trial topic’/exp OR ‘randomized controlled trial topic’ OR ‘retrospective study’/exp OR ‘retrospective study’ OR ‘systematic review’/exp OR ‘systematic review’) AND ‘article’/it	225

### Appendix A.3. Scopus

**Set #**	**Concept**	**Syntax**	**Results**
1	Alzheimer spectrum (population)	TITLE-ABS-KEY(Alzheimer * OR “AD dementia” OR “mild cognitive impairment” OR MCI)	375,217
2	Metabolic & bioenergetic dysfunction	TITLE-ABS-KEY(brain metabolism OR glucose hypometabolism OR insulin resistance OR mitochondrial dysfunction OR oxidative stress)	2154
3	Imaging (PET)	TITLE-ABS-KEY(PET OR FDG-PET OR 18F-FDG)	270,972
4	Amyloid pathology	TITLE-ABS-KEY(“amyloid beta” OR amyloid accumulation OR amyloid deposition)	21,833
5	Tau pathology	TITLE-ABS-KEY(tau OR “tau pathology” OR neurofibrillary tangle *)	20,641
6	Limits/Filters	English; Publication year ≥ 2021; Article only; Medicine/Neuroscience	
7	Full combined query	TITLE-ABS-KEY (Alzheimer * OR “AD dementia” OR “mild cognitive impairment” OR MCI OR “prodromal Alzheimer *”) AND TITLE-ABS-KEY (“brain metabolism” OR “cerebral metabolism” OR “cerebral glucose metabolism” OR “brain glucose” OR hypometabolism OR “glucose hypometabolism” OR “glucose hypometaboli *” OR “energy failure” OR “brain energy failure” OR “energy metabolism failure” OR “metabolic dysfunction” OR “metabolic dysfunct *” OR “insulin resistance” OR “brain insulin resistance” OR “central insulin resistance” OR “insulin receptor *” OR “brain insulin receptor *” OR “type 3 diabetes” OR mitochondria * OR “mitochondrial dysfunction” OR “mitochondrial bioenergetics” OR “ATP depletion” OR “oxidative stress” OR glutathione OR “reactive oxygen species” OR ROS OR “redox imbalance”) AND TITLE-ABS-KEY (PET OR “FDG-PET” OR “18F-FDG” OR fluorodeoxyglucose) AND TITLE-ABS-KEY (“amyloid beta” OR Abeta OR Aβ OR “amyloid accumulation” OR “amyloid deposition”) AND TITLE-ABS-KEY (tau OR “tau pathology” OR “neurofibrillary tangle *” OR “paired helical filament *”) AND PUBYEAR > 2019 AND PUBYEAR < 2027 AND (LIMIT-TO (PUBSTAGE, “final”) OR LIMIT-TO (PUBSTAGE, “aip”)) AND (LIMIT-TO (DOCTYPE, “ar”)) AND (LIMIT-TO (SUBJAREA, “MEDI”) OR LIMIT-TO (SUBJAREA, “NEUR”)) AND (LIMIT-TO (LANGUAGE, “English”)) AND (LIMIT-TO (EXACTKEYWORD, “Alzheimer Disease”) OR LIMIT-TO (EXACTKEYWORD, “Humans”))	189
